# Supplementary material for: Divergent CRD-Dependent Mechanisms Govern RAS Isoform-Selective Recruitment of CRAF and ARAF
Source: bioRxiv. 2026 May 11:2026.05.08.723844. Preprint. [Version 1] doi: 10.64898/2026.05.08.723844 (PMC13192977; doi:10.64898/2026.05.08.723844)
Supplement: Supplement 1 [file media-1.pdf]

Divergent CRD-Dependent Mechanisms Govern RAS Isoform-Selective Recruitment of CRAF and ARAF

Shrhea Banerjee, Sravani Malasani, Shriti Banerjee, Maria Celeste López Vásquez, Shane McSorley, and Zhihong Wang*

Department of Chemistry & Biochemistry, Rowan University, Glassboro, NJ

Supplementary Information

- Supplementary Figures 1-7
- Supplementary Table 1 and 2

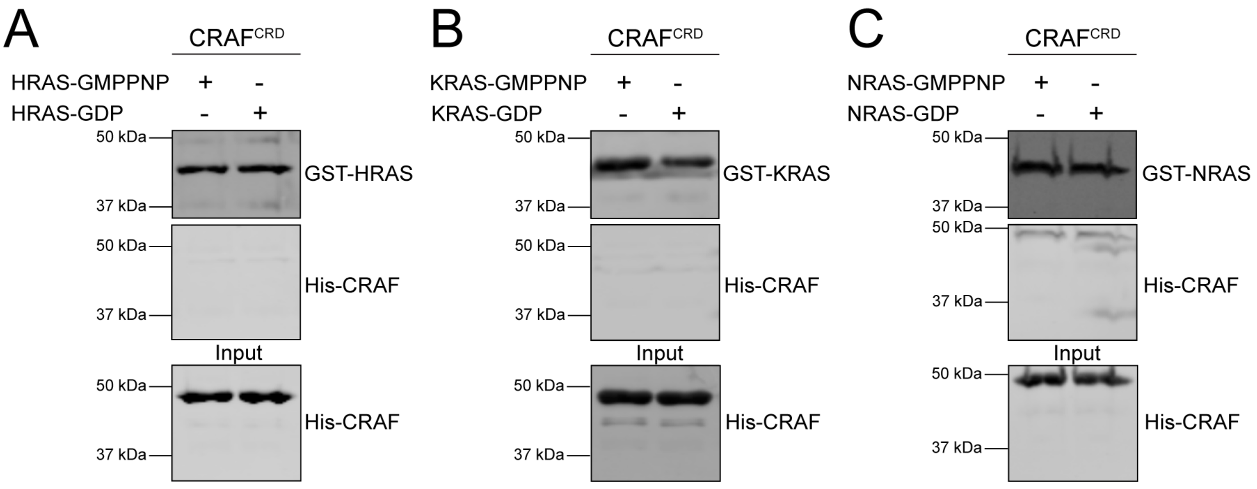

Figure S1. The isolated CRAF^{CRD} is insufficient for stable interaction with RAS isoforms. A-C) GST pulldown assays assessing binding of the isolated His-tagged CRAF^{CRD} to HRAS (A), KRAS (B), or NRAS (C) loaded with either GMPPNP or GDP. Pulldown experiments were performed in two independent replicates with comparable results.

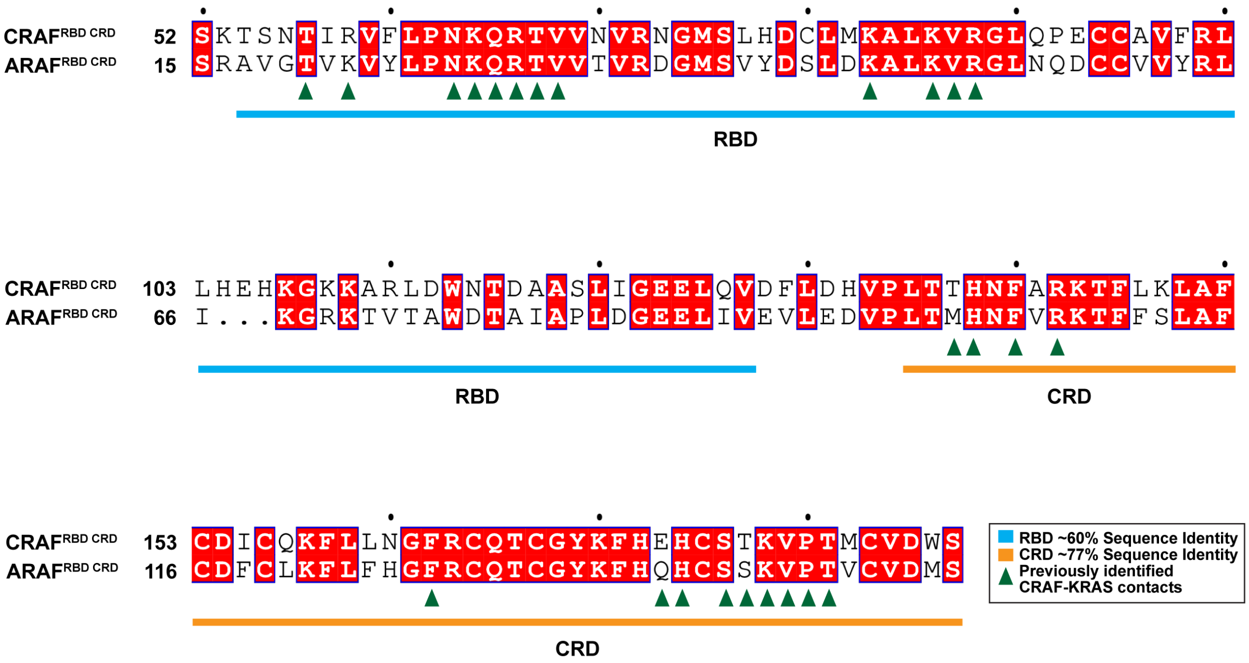

Figure S2. Sequence alignment of CRAF and ARAF RBD and CRD domains highlighting conservation and RAS-interacting residues. Pairwise sequence alignment of the RBD and CRD of human CRAF and ARAF. Residues are colored according to sequence identity (red shading), with identical residues highlighted. Domain boundaries for the RBD (blue) and CRD (orange) are indicated below the alignment. CRAF residues previously identified to participate in KRAS interaction are marked (green triangles).

A

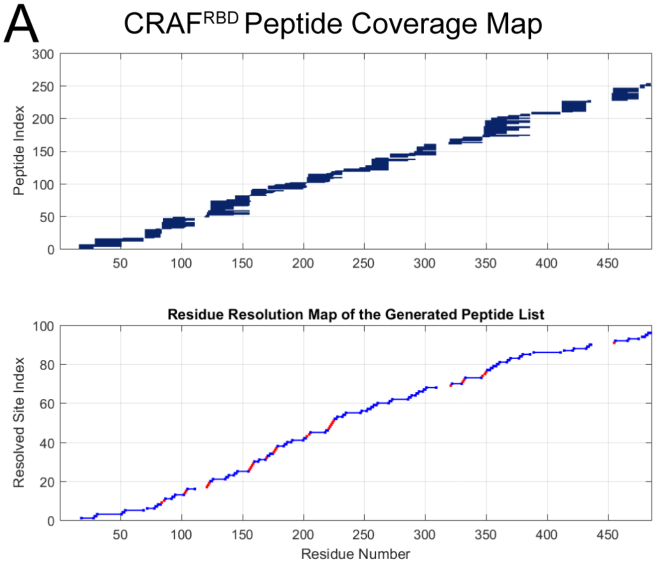

B

CRAF^{RBD} Sequence:
1- HHHHHHGTKTEEGKLVIWINGDKGYNGLAEVGKKFEKDTGIKVTVEHPDKL
EEKFPQVAATGDGPDIIFWAHDRFGGYAQSGLLAEITPDKAFQDKLYPFTWDA
VRYNGKLIAYPIAVEALSLIYNKDLLPNPPKTWEEIPALDKELKAKGKSALMFNL
QEPYFTWPLIAADGGYAFKYENGKYDIKDVGVDNAGAKAGLTFLVDLIKNKHM
NADTDYSIAEAAFNKGETAMTINGPWAWSNIDTSKVNYGVTVLPTFKGQPSKP
FVGVLSAGINAASPNKELAKEFLENYLLTDEGLEAVNKDKPLGAVALKSYEEEL
AKDPRIAATMENAQKGEIMPNIPQMSAFWYAVRTAVINAASGRQTVDEALKDA
QTGTDYDIPTTENLYFQGHMASMTGGQQMGRGSSKTSNTIRVFLPNKQRTV
VNVRNGMSLHDCLMKALKVRGLQPECCAVFRLLHEHKGKKARLDWNTDAAS
LIGEELQVDFL -485

His/MBP tag
CRAF^{RBD} (a.a. 52-131)

Figure S3. Peptide coverage and sequence representation of the CRAF^{RBD} used for HDX-MS analysis. A) Stripe plots depicting peptide coverage across the CRAF^{RBD}, showing identified peptides (top) and the corresponding residue resolution map generated from the peptide list (bottom). B) Amino acid sequence of the CRAF^{RBD} construct used for HDX-MS, with the region analyzed highlighted in purple and the N-terminal His/MBP tag indicated in green. Residue numbering corresponds to the full-length CRAF sequence.

A

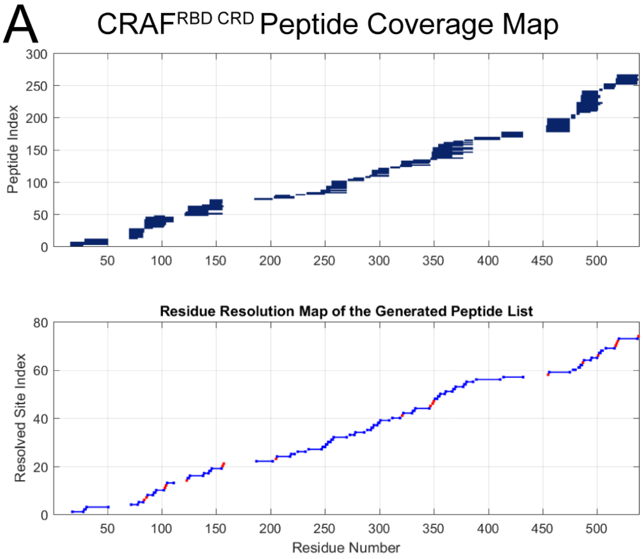

B

CRAF$^{\mathrm{RBD\ CRD}}$ Sequence:
1- HHHHHHGTKTEEGKLVIWINGDKGYNGLAEVGKKFEKDTGIKVTVEHPDKL
EEKFPQVAATGDGPDIIFWAHDRFGGYAQSGLLAEITPDKAFQDKLYPFTWDA
VRYNGKLIAYPIAVEALSLIYNKDLLPNPPKTWEEIPALDKELKAKGKSALMFNL
QEPYFTWPLIAADGGYAFKYENGKYDIKDVGVDNAGAKAGLTFLVDLIKNKHM
NADTDYSIAEAAFNKGETAMTINGPWAWSNIDTSKVNYGVTVLPTFKGQPSK
PFVGVLSAGINAASPNKELAKEFLENYLLTDEGLEAVNKDKPLGAVALKSYEEE
LAKDPRIAATMENAQKGEIMPNIPQMSAFWYAVRTAVINAASGRQTVDEALKD
AQTGTDYDIPTTENLYFQGHMASMTGGQQMGRGSSKTSNTIRVFLPNKQRT
VVNVRNGMSLHDCLMKALKVRGLQPECCAVFRLLHEHKGKKARLDWNTDA
ASLIGEELQVDFLDHVPLTTHNFARKTFLKLAFCDICQKFLLNGFRCQTCGYKF
HEHCSTKVPTMCVDWS -542

His/MBP tag
CRAF$^{\mathrm{RBD\ CRD}}$ (a.a. 52-188)

Figure S4. Peptide coverage and sequence representation of the CRAF$^{\mathrm{RBD\ CRD}}$ used for HDX-MS analysis. A) Stripe plots depicting peptide coverage across the CRAF$^{\mathrm{RBD\ CRD}}$, showing identified peptides (top) and the corresponding residue resolution map generated from the peptide list (bottom). B) Amino acid sequence of the CRAF$^{\mathrm{RBD\ CRD}}$ construct used for HDX-MS, with the region analyzed highlighted in brown and the N-terminal His/MBP tag indicated in green. Residue numbering corresponds to the full-length CRAF sequence.

A

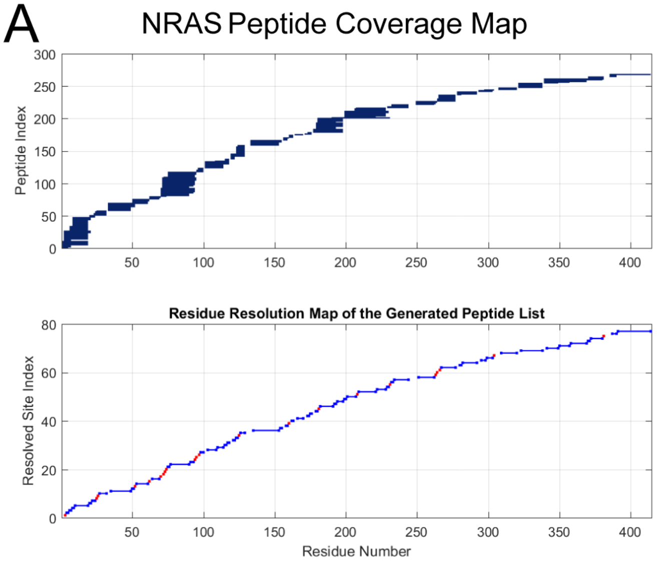

B

NRAS Sequence:
1- MSPILGYWKIKGLVQPTRLLLEYLEEKYEEHLYERDEGDKWRNKKFELGLE
FPNLPYYIDGDVKLTQSMAIIRYIADKHNMLGGCPKERAEISMLEGAVLDIRYG
VSRIAYSKDFETLKVDFLSKLPEMLKMFEDRLCHKTYLNGDHVTHPDFMLYDA
LDVVLYMDPMCLDAFPKLVCFKKRIEAIPQIDKYLKSSKYIAWPLQGWQATFGG
GDHPPKSDLVPRGSMTEYKLVVVGAGGVGKSALTIQLIQNHFVDEYDPTIEDSY
RKQVVIDGETCLLDILDTAGQEEYSAMRDQYMRTGEGFLCVFAINNSKSFADI
NLYREQIKRVKDSDDVPMVLVGNKCDLPTRTVDTKQAHELAKSYGIPFIETSAK
TRQGVEDAFYTLVREIRQYRMKKLNSSDDGTQGCMGLPCVVM -414

GST tag
NRAS (a.a. 1-189)

Figure S5. Peptide coverage and sequence representation of NRAS used for HDX-MS analysis. A) Stripe plots depicting peptide coverage across NRAS, showing identified peptides (top) and the corresponding residue resolution map generated from the peptide list (bottom). B) Amino acid sequence of NRAS construct used for HDX-MS, with the region analyzed highlighted in pink and the N-terminal GST tag indicated in green.

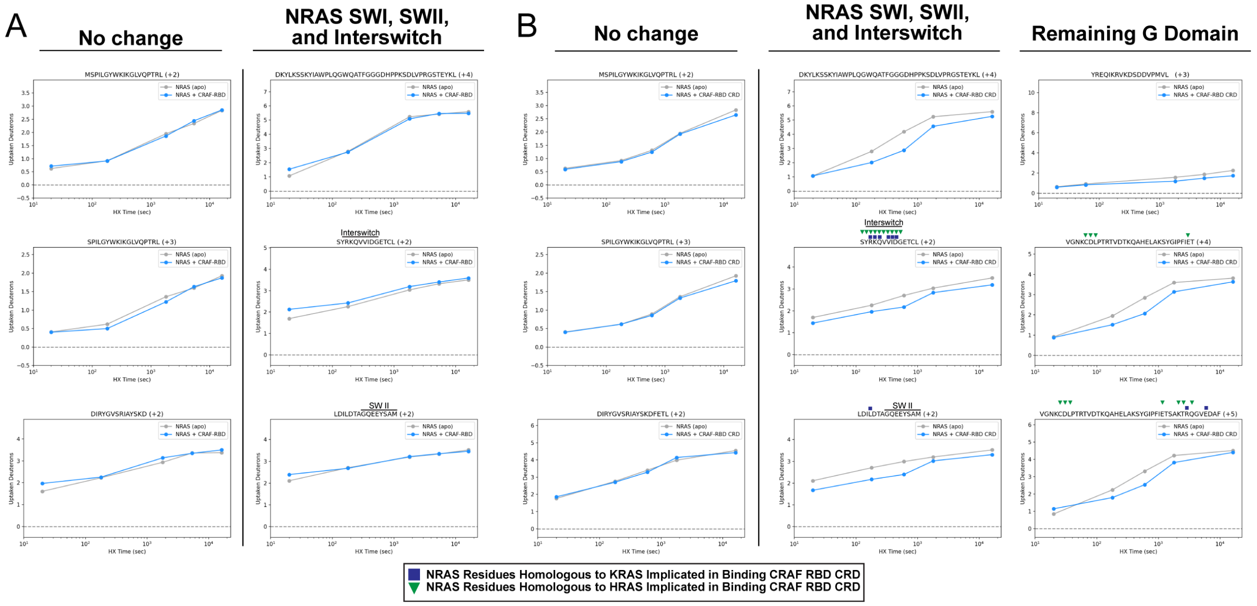

Figure S6. Reciprocal HDX-MS analysis comparing apo NRAS with NRAS bound to CRAF^{RBD} or CRAF$^{RBD\ CRD}$. A) Representative deuterium uptake plots for NRAS peptides exhibiting no significant change in exchange in the presence of CRAF^{RBD}. B) Deuterium uptake plots for peptides spanning NRAS SWI, SWII, interswitch regions, and additional regions within the remaining G-domain that display reduced deuterium uptake in the presence of CRAF$^{RBD\ CRD}$, resulting in reproducible reductions in deuterium incorporation consistent with stabilization of the effector-binding interface. NRAS residues homologous to KRAS (blue squares) and HRAS (green triangle) residues previously implicated in binding CRAF$^{RBD\ CRD}$ have been indicated.

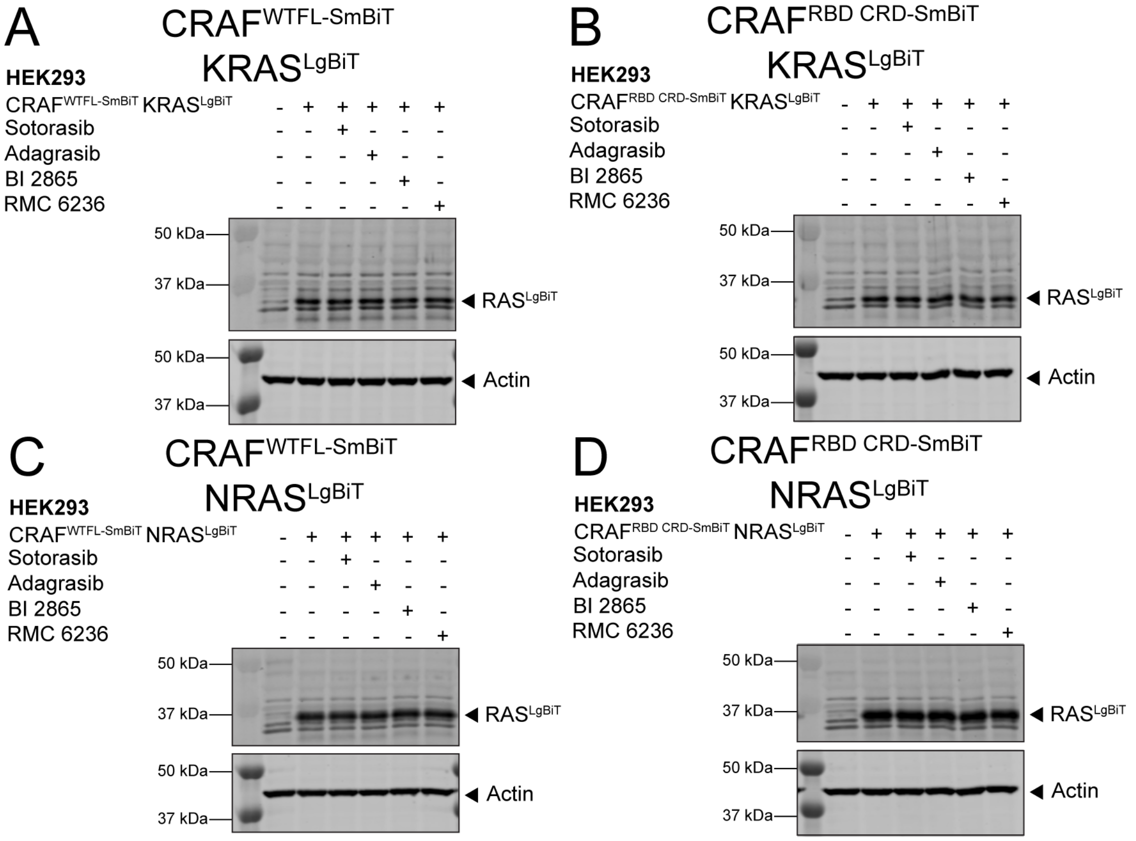

Figure S7. RAS inhibitors disrupt RAS–CRAF interactions without altering protein expression in cells. A-B) Representative immunoblots of HEK293 cells expressing CRAF$^{WTFL\text{-}SmBiT}$ with KRAS^{LgBiT} (A) or CRAF$^{RBD\ CRD\text{-}SmBiT}$ with KRAS^{LgBiT} (B) in the absence or presence of the indicated RAS inhibitors (Sotorasib, Adagrasib, BI 2865, and RMC 6236). C-D) Immunoblots of HEK293 cells expressing CRAF$^{WTFL\text{-}SmBiT}$ with NRAS^{LgBiT} (C) or CRAF$^{RBD\text{-}CRD\text{-}SmBiT}$ with NRAS^{LgBiT} (D) under the same treatment conditions.

Table S1. BLI Kinetic Data Summary

Binding Partners	K_D (nM)	K_{on} (1/Ms)	K_{off} (1/s)	X^2	R^2
$CRAF^{RBD}$-HRAS	63.23	1.155×10^4	7.303×10^{-4}	3.65	0.993
	40.24	2.455×10^4	9.880×10^{-4}	0.43	0.997
$CRAF^{RBD}$-KRAS	96.66	3.107×10^4	3.004×10^{-3}	1.90	0.997
	101.77	4.926×10^4	5.012×10^{-3}	3.46	0.992
$CRAF^{RBD}$-NRAS	325.7	1.137×10^4	3.704×10^{-3}	1.64	0.987
	398.0	1.154×10^4	4.595×10^{-3}	1.54	0.990
$CRAF^{RBD\ CRD}$-HRAS	61.15	2.458×10^4	1.503×10^{-3}	0.70	0.987
	51.17	6.272×10^4	3.210×10^{-3}	0.94	0.994
$CRAF^{RBD\ CRD}$-KRAS	30.31	1.040×10^3	3.154×10^{-5}	3.11	0.992
	34.80	1.390×10^3	4.838×10^{-5}	2.74	0.971
$CRAF^{RBD\ CRD}$-NRAS	73.41	2.452×10^4	1.800×10^{-3}	0.51	0.994
	74.88	6.832×10^4	5.116×10^{-3}	1.14	0.989
$ARAF^{RBD}$-HRAS	2493	9.507×10^2	2.370×10^{-3}	0.66	0.981
	6838	3.684×10^2	2.519×10^{-3}	2.24	0.978
$ARAF^{RBD}$-KRAS	966.65	3.068×10^2	2.966×10^{-4}	0.69	0.971
	1519	2.950×10^2	4.481×10^{-4}	0.25	0.983
$ARAF^{RBD}$-NRAS	8289	1.007×10^3	1.575×10^{-3}	0.63	0.975
	6082	1.007×10^3	6.122×10^{-3}	1.60	0.984
$ARAF^{RBD\ CRD}$-HRAS	7294	4.866×10^2	3.550×10^{-3}	0.28	0.980
	11981	2.367×10^2	2.836×10^{-3}	0.92	0.918
$ARAF^{RBD\ CRD}$-KRAS	1888	9.619×10^2	1.816×10^{-3}	0.20	0.964
	4449	2.193×10^2	9.757×10^{-4}	1.49	0.945
$ARAF^{RBD\ CRD}$-NRAS	1227	4.291×10^2	5.267×10^{-4}	0.66	0.990
	4268	3.477×10^2	1.484×10^{-3}	2.45	0.978
$CRAF^{RBD\ CRD}$-$CRAF^{KD}$	47.53	1.580×10^6	7.509×10^{-2}	0.02	0.994
	39.72	1.181×10^6	4.691×10^{-2}	0.10	0.976
$CRAF^{RBD\ CRD}$+KRAS-$CRAF^{KD}$	600.16	1.295×10^5	7.772×10^{-2}	0.06	0.945
	519.91	1.377×10^5	7.160×10^{-2}	0.24	0.894
$CRAF^{RBD\ CRD}$-KRAS+BI2865	713.77	1.114×10^4	7.953×10^{-3}	1.17	0.945
	421.87	7.406×10^4	2.145×10^{-4}	0.70	0.957
$CRAF^{RBD\ CRD}$-KRAS+RMC6236	1901	3.986×10^4	7.577×10^{-2}	1.73	0.947
	1496	5.304×10^4	7.935×10^{-2}	0.38	0.983
$CRAF^{RBD\ CRD}$-NRAS+RMC6236	633.54	6.617×10^4	4.192×10^{-2}	0.40	0.991
	614.45	5.554×10^4	3.413×10^{-2}	0.46	0.97

Table S2. List of Previously Published K_D Values

RAF Construct	RAS Construct	Binding Affinity (nM)	Method	Expression System	Reference
$CRAF^{RBD}$ (51-131)	HRAS	270 ± 90	Total internal reflection fluorescence (TIRF) microscopy (2% PS)	E.coli	(Jimenez Salinas et al., 2026)
$CRAF^{RBD}$ (51-131)	HRAS	240 ± 20	TIRF (20% PS)	E.coli	(Jimenez Salinas et al., 2026)
$CRAF^{RBD\,CRD}$ (51-188)	HRAS	25 ± 10	TIRF (2% PS)	E.coli	(Jimenez Salinas et al., 2026)
$CRAF^{RBD\,CRD}$ (51-188)	HRAS	6 ± 3	TIRF (20% PS)	E.coli	(Jimenez Salinas et al., 2026)
$CRAF^{CRD}$ (138-188)	HRAS	NB	TIRF (20% PS)	E.coli	(Jimenez Salinas et al., 2026)
$CRAF^{RBD}$ (51-131)	HRAS	220 ± 80	TIRF (PEGylated)	E.coli	(Jimenez Salinas et al., 2026)
$CRAF^{RBD\,CRD}$ (51-188)	HRAS	40 ± 10	TIRF (PEGylated)	E.coli	(Jimenez Salinas et al., 2026)
$CRAF^{RBD}$ (52-131)	KRAS (1-169)	356	Surface Plasmon Resonance (SPR)	E.coli	(Tran et al., 2021)
$CRAF^{RBD\,CRD}$ (52-192)	KRAS (1-169)	152	SPR	E.coli	(Tran et al., 2021)
$CRAF^{RBD}$ (51-131)	KRAS (1-166)	98 ± 2	Isothermal Titration Calorimetry (ITC)	E.coli	(Johnson et al., 2017)

$CRAF^{RBD}$ (51-131)	HRAS (1-166)	94 ± 4	ITC	E.coli	(Johnson et al., 2017)
$CRAF^{RBD}$ (51-131)	NRAS (1-166)	206 ± 8	ITC	E.coli	(Johnson et al., 2017)
$CRAF^{RBD}$ (1-149)	HRAS (farnesylated)	24.3	SPR	E.coli	(Fischer et al., 2007)
$CRAF^{RBD}$ (1-149)	$HRAS^{G12V}$ (farnesylated)	21.3	SPR	E.coli	(Fischer et al., 2007)
$CRAF^{RBD}$ (1-149)	HRAS	21.2	SPR	E.coli	(Fischer et al., 2007)
$BRAF^{RBD}$ (1-245)	HRAS	11.2	SPR	E.coli	(Fischer et al., 2007)
$ARAF^{RBD}$	HRAS	76.6	SPR	E.coli	(Fischer et al., 2007)
$CRAF^{FL}$	HRAS (farnesylated)	460	SPR	HRAS: E.coli CRAF: Sf9	(Fischer et al., 2007)
$CRAF^{R89L}$	HRAS (farnesylated)	860	SPR	HRAS: E.coli CRAF: Sf9	(Fischer et al., 2007)
$CRAF^{FL}$	HRAS	NB	SPR	HRAS: E.coli CRAF: Sf9	(Fischer et al., 2007)
$BRAF^{FL}$	HRAS (farnesylated)	58.8	SPR	HRAS: E.coli BRAF: Sf9	(Fischer et al., 2007)
$BRAF^{FL}$	HRAS	80.2	SPR	HRAS: E.coli BRAF: Sf9	(Fischer et al., 2007)
$BRAF^{\Delta98}$ (99-766)	HRAS (farnesylated)	75.6	SPR	HRAS: E.coli BRAF: Sf9	(Fischer et al., 2007)
$BRAF^{\Delta98}$ (99-766)	HRAS	290.4	SPR	HRAS: E.coli BRAF: Sf9	(Fischer et al., 2007)
$CRAF^{RBD}$ CRD (1-192)	HRAS (farnesylated)	122.3	SPR	E.coli	(Fischer et al., 2007)
$CRAF^{RBD}$ CRD	HRAS	190.5	SPR	E.coli	(Fischer et al., 2007)

(1-192)					
$CRAF^{N-terminal}$ (1-303)	HRAS (farnesylated)	104.6	SPR	E.coli	(Fischer et al., 2007)
$CRAF^{N-terminal}$ (1-303)	HRAS	188	SPR	E.coli	(Fischer et al., 2007)
$BRAF^{N-terminal}$ (1-412)	HRAS (farnesylated)	62.4	SPR	E.coli	(Fischer et al., 2007)
$BRAF^{N-terminal}$ (1-412)	HRAS	64.4	SPR	E.coli	(Fischer et al., 2007)
$CRAF^{RBD}$ (51-131)	Ras·mantGppNHp	160	SPR	E.coli	(Sydor et al., 1998)
$CRAF^{RBD}_{L91W/H}$ (51-131)	H + Ras·mantGppNHp	780	SPR	E.coli	(Sydor et al., 1998)
$CRAF^{RBD}$ (51-131)	Ras·mantGppNHp	300	SPR	E.coli	(Sydor et al., 1998)
$CRAF^{RBD}_{L91W/H}$ (51-131)	H + Ras·GppNHp	380	SPR	E.coli	(Sydor et al., 1998)
$CRAF^{RBD}$ (51-131)	Ras·mantGDP	32,000	SPR	E.coli	(Sydor et al., 1998)

References

Fischer, A., Hekman, M., Kuhlmann, J., Rubio, I., Wiese, S., & Rapp, U. R. (2007). B- and C-RAF Display Essential Differences in Their Binding to Ras: THE ISOTYPE-SPECIFIC N TERMINUS OF B-RAF FACILITATES RAS BINDING*. *Journal of Biological Chemistry*, *282*(36), 26503-26516. https://doi.org/10.1074/jbc.M607458200

Jimenez Salinas, A., Tevdorashvili, K., Grim, J., Morales, A., Chakhrakia, A., & Kwang Lee, Y. (2026). Positive cooperativity between RAS-binding and cysteine-rich domains regulates RAF membrane binding kinetics via lateral rebinding. *Nature Communications*. https://doi.org/10.1038/s41467-026-69437-6

Johnson, C. W., Reid, D., Parker, J. A., Salter, S., Knihtila, R., Kuzmic, P., & Mattos, C. (2017). The small GTPases K-Ras, N-Ras, and H-Ras have distinct biochemical properties determined by allosteric effects. *Journal of Biological Chemistry*, *292*(31), 12981-12993. https://doi.org/10.1074/jbc.M117.778886

Sydor, J. R., Engelhard, M., Wittinghofer, A., Goody, R. S., & Herrmann, C. (1998). Transient Kinetic Studies on the Interaction of Ras and the Ras-Binding Domain of c-Raf-1 Reveal Rapid Equilibration of the Complex. *Biochemistry*, *37*(40), 14292-14299. https://doi.org/10.1021/bi980764f

Tran, T. H., Chan, A. H., Young, L. C., Bindu, L., Neale, C., Messing, S., Dharmaiah, S., Taylor, T., Denson, J.-P., Esposito, D., Nissley, D. V., Stephen, A. G., McCormick, F., & Simanshu, D. K. (2021). KRAS interaction with RAF1 RAS-binding domain and cysteine-rich domain provides insights into RAS-mediated RAF activation. *Nature Communications*, *12*(1), 1176. https://doi.org/10.1038/s41467-021-21422-x
